# Introduction of total knee arthroplasty in Lithuania: Results from the first 10 years

**DOI:** 10.1080/17453670902804984

**Published:** 2009-02-01

**Authors:** Sarunas Tarasevicius, Justinas Stucinskas, Otto Robertsson, Hans Wingstrand

**Affiliations:** ^1^Department of Orthopedics, Kaunas Medical UniversityKaunas, LithuaniaSweden; ^2^Department of Orthopedics, Lund University HospitalLundSweden

## Abstract

**Background and purpose** We have previously reported that the first 10 years of hip arthroplasty in Lithuania resulted in a higher cumulative revision rate than that observed in Sweden. We thus compared the corresponding results after introduaction of total knee replacement in Lithuania.

**Methods** The 10-year revision rate for the first 595 primary ScanKnee arthroplasties inserted in Klaipeda, Lithuania, was compared to that for the first 1,280 ScanKnee primary arthroplasties inserted in Sweden. As in the hip replacement study, only patients with osteoarthritis (OA) were included. Primary knee arthroplasties without patellar resurfacing were included, and the endpoint was revision for any reason other than addition of a patellar component.

**Results** We found that the cumulative revision rate was not statistically significantly different between the groups. The revision pattern was different, however, and we observed 24 isolated patellar component additions in Sweden, but none in Klaipeda.

**Interpretation** Contrary to the results of our previous hip arthroplasty study, the cumulative revision rate after total knee arthroplasty was similar in the two groups. This suggests that compared to hip arthroplasty, the outcome of total knee arthroplasty was less dependent on surgical experience. The large difference regarding isolated patellar component additions may be explained by long-term accumulation of severe OA cases in Lithuania. To patients subject to a newly introduced surgical treatment offering great improvement in quality of life, patellofemoral pain may be a minor problem. Furthermore, patellar problems may not have seemed particularly relevant for the surgeons, considering the disability of other patients waiting to be treated.

## Introduction

In a previous study we compared the cumulative revision rates (CRRs) after the introduction of total hip arthroplasty (THA) in Lithuania to that observed in Sweden with its previous experience of this type of surgery (Tarasevicius et al. 2007). We found that the CRR was 12% in Lithuania and 6% in Sweden (p = 0.003). Total knee arthroplasty (TKA) was introduced in Lithuania in 1993, 2 years later than THA, and we felt it relevant to study whether the outcome after the introduction of TKA was also different.

In 1993, when the ScanKnee implant was selected to be used in Klaipeda, modern knee arthroplasty surgery was new in Lithuania. As in our previous study, all TKAs were followed-up prospectively from the beginning. In Sweden, the same ScanKnee implant had been introduced in 1987. Unlike in Klaipeda, however, there was ample previous surgical experience regarding the use of TKA.

We compared the CRR in Klaipeda with that in Sweden during the first 10 years that the same knee implant was in use in both locations.

## Patients and methods

Only OA patients with a primary ScanKnee without patellar resurfacing were included. From 1993 to 1998, this implant was the only one used in Klaipeda. 595 knees were implanted in 503 patients from August 1993 to December 2002. In Sweden, the ScanKnee implant was introduced in 1987 and 1,280 implants in 1,176 patients had been inserted between 1987 and 1996, at 26 hospitals all over the country (Table 1). The Swedish patients were followed-up until 2001 and the Klaipeda patients until 2007. Thus, as in the previous THA study, the follow-up period ranged from 3 to 14 years in both groups.

**Table 1.** Data on TKR patients in Klaipeda (Lithuania) and Sweden

Country	A	B	C	D	E
Sweden	1,280	885	59	320	73 [7.5] (33–94)
Klaipeda	595	519	24	60	68 [7.2] (37–85)

A: Total number

B: Female patients

C: Revised

D: Dead, unrevised

E: Mean age [SD] (range)

2 orthopedic surgeons who had had a short training period in Sweden, but otherwise no experience of knee arthroplasty, inserted all the implants in Klaipeda. We have no information about the number of Swedish surgeons involved; the Swedish data were obtained from the Swedish Knee Arthroplasty Register (SKAR), which does not register the name of the surgeon.

All the Klaipeda patients, and probably most of the Swedish patients, had an anterior incision and a medial arthrotomy. The cement initially used in Klaipeda was the CMW I with gentamicin, but in the year 2000 it was replaced by the SmartSet with gentamicin. The cementing technique in Klaipeda was hand mixing and no pulsating lavage. Almost all operations in Sweden were performed with vacuum-mixed Palacos cement with gentamicin and pulsating lavage to clean the bone.

In Klaipeda, information about revisions possibly performed elsewhere in Lithuania was obtained from the 2 other orthopedic centers performing revisions. The Lithuanian State Patients Fund provided information about deceased patients. All the Swedish data were obtained from the SKAR, which prospectively gathers information about primary arthroplasties and subsequent revisions, and deaths.

Revision was defined as addition, exchange, or removal of one or both components. We analyzed CRR using all revisions as an endpoint after excluding revisions in which only a patellar button had been added.

### Statistics

CRR curves were produced using the life table method at monthly intervals. Curves were cut when 40 knees remained at risk. 95% confidence intervals (CIs) were calculated, using the Wilson quadratic equation with Greenwood and Peto effective sample-size estimates (Dorey et al. 1993). The Wilcoxon test was used when comparing the difference in revision rate between the two groups. Additionally, Cox regression was used to adjust for differences in age and sex. The age of the patient at the primary operation was used as continuous variable and its ratio expresses the change in risk for each year the age increases. The location and sex were categorical variables in which Klaipeda was a reference for Sweden and male sex was a reference for female sex. In the study, it was possible for both knees of the same patient to become included, which might raise the question of bias—as by definition bilateral observations are not independent. However, we felt that this was permissible as the problem has been investigated with respect to survival after both knee arthroplasty (Robertsson et al. 2003) and hip arthroplasty (Lie et al. 2004), and was not considered to be an issue.

A p-value of < 0.05 was considered to be statistically significant. SPSS version 15.0 and Microsoft Excel software were used for the calculations.

## Results

**Figure 1.**
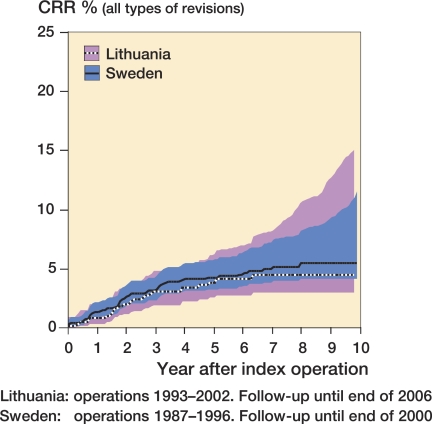
Cumulative revision rate (all types of revisions) with 95% confidence intervals for Klaipeda and Sweden; p = 0.56 (Wilcoxon test).

**Figure 2.**
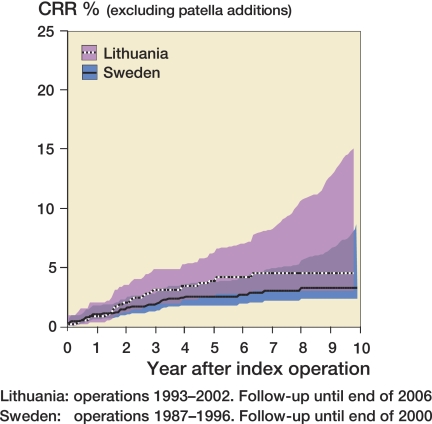
Cumulative revision rate (excluding patella additions) with 95% confidence intervals for Klaipeda and Sweden; p = 0.15 (Wilcoxon test).

When comparing the cumulative revision rates in Klaipeda with those in Sweden, we found no statistically significant difference (Figures 1 and 2).

Except for patellar resurfacing in Sweden, aseptic loosening was the commonest reason for revision (Table 2). Using any revision as endpoint, the overall CRR after 10 years was 4.5% (95% CI: 3.0–16) for the Klaipeda patients and 5.5% (95% CI: 4.2–12) for the Swedish patients (Figure 1). The difference was not statistically significant (p = 0.6, Wilcoxon). Cox regression analysis, adjusting for age and sex, showed that the risk of revision decreased with increasing age, while sex and country had no statistically significant effect on the revision rate (Table 3).

**Table 2.** Breakdown of types of revision

Reason for revision	Sweden n=59	Klaipeda n=24
Loosening	12	12
Wear	4	0
Infection	9	7
Instability	2	2
Malposition	0	3
Fracture	1	0
Patella	24	0
Pain NUD	4	0
Other	3	0

**Table 3.** Cox regression data. Lithuania is used as reference for Sweden, and males are used as reference for females

	RR	95% CI	p-value
All revisions			
Age	0.94	0.92–0.97	< 0.001
Location	1.55	0.94–2.57	0.08
Sex	1.09	0.65–1.81	0.8
Patella revisions excluded			
Age	0.95	0.92–0.97	< 0.001
Location	0.91	0.52–1.59	0.7
Sex	1.15	0.61–2.16	0.7

Revisions in which a patellar component was added were not performed in Klaipeda whereas there were 24 in Sweden, representing 40% of the Swedish revisions. If these were excluded, the overall CRR in Sweden after 10 years fell to 3.3% (CI: 2.3–8.6). However, the difference was not statistically significant (p = 0.6, Wilcoxon). When adjustment was made for age and sex by Cox regression, the results for age and sex remained practically unchanged. While the risk ratio for Sweden became lower than for Klaipeda, the difference was not statistically significant (Table 3).

## Discussion

We found no difference in the overall cumulative revision rate (CCR) when comparing ScanKnee implants inserted in Klaipeda, Lithuania, to those inserted in Sweden, regardless of whether Swedish patients who were reoperated with only patellar additions were excluded or not. This differed from our findings with THA, in which Klaipeda had a 6% higher revision rate. Thus, it seems that TKA is less sensitive to lack of prior arthroplasty experience than THA.

The types of cement were different in the groups. In Klaipeda CMW1 and SmartSet with gentamicin was used, while Palacos with gentamicin was used in Sweden. Compared to Palacos cement, CMW 1 has been shown to have a higher revision rate in THA (Espehaug et al. 2002). However, to our knowledge the type of cement has not been reported to affect long-term outcome after TKA.

There were differences in the cementing technique between countries. In Klaipeda the cement was hand-mixed and no pulsative jet lavage was used, while in Sweden a vacuum cement mixing system and pulsative jet lavage were used in most cases. Ritter et al. (1994) reported more radiolucency around the tibial components of knees that had been prepared without pulsative lavage. Maistrelli et al. (1995) found a statistically significant increase in cement penetration in specimens that were washed with pulsative lavage than those washed with simple irrigation. Thus, it seems that pulsative jet lavage and vacuum mixing of the cement improves cement-bone fixation. However, although modern cementing technique has been shown to lower CRR in THA (Herberts and Malchau 2000), we are not aware of any reports claiming similar improvement after TKA.

In Sweden, 24 reoperations were performed with only a patellar component being added. No such revisions were performed in Lithuania. These revisions may be considered minor revisions, and there are some possible explanations as to why they were common in Sweden but not in Lithuania. The main reason is probably the different populations of patients. As there were limited surgical options before the introduction of arthroplasty in Lithuania, there had been an accumulation of untreated advanced cases. It is likely that in patients with severe joint destruction, contractures, and deformities, the improvement after a TKA—with respect to pain and function—is so great that moderate anterior knee pain becomes insignificant. The opposite is probably true for patients with less severe OA, in whom maximum function and pain relief is expected. Furthermore, as there has been no tradition of resurfacing the patella in primary cases in Lithuania, the surgeons may be more reluctant to offer it as a secondary treatment.

We found that the CRR after TKA was similar in Lithuania and Sweden, which is contrary to our previous findings in patients after THA (Tarasevicius et al. 2007). This suggests that the introduction of TKA may be less complicated than that of THA.

## References

[CIT0001] Dorey F, Nasser S, Amstutz H (1993). The need for confidence intervals in the presentation of orthopaedic data.. J Bone Joint Surg (Am).

[CIT0002] Espehaug B, Havelin LI, Engesaeter LB, Vollset SE (1999). The effect of hospital-type and operating volume on the survival of hip replacements. A review of 39,505 primary total hip replacements reported to the Norwegian Arthroplasty Register, 1988-1996.. Acta Orthop Scand.

[CIT0003] Espehaug B, Furnes O, Havelin LI, Engesaeter LB, Vollset SE (2002). The type of cement and failure of total hip replacements.. J Bone Joint Surg (Br).

[CIT0004] Herberts P, Malchau H (2000). Long-term registration has improved the quality of hip replacement: a review of the Swedish THR Register comparing 160,000 cases.. Acta Orthop Scand.

[CIT0005] Lie SA, Engesaeter LB, Havelin LI, Gjessing HK, Vollset SE (2004). Dependency issues in survival analyses of 55,782 primary hip replacements from 47,355 patients.. Stat Med.

[CIT0006] Maistrelli GL, Antonelli L, Fornasier V, Mahomed N (1995). Cement penetration with pulsed lavage versus syringe irrigation in total knee arthroplasty.. Clin Orthop.

[CIT0007] Ritter MA, Herbst SA, Keating EM, Faris PM (1994). Radiolucency at the bone-cement interface in total knee replacement. The effects of bone-surface preparation and cement technique.. J Bone Joint Surg (Am).

[CIT0008] Robertsson O, Ranstam J (2003). No bias of ignored bilaterality when analysing the revision risk of knee prostheses: Analysis of a population based sample of 44,590 patients with 55,298 knee prostheses from the national Swedish Knee Arthroplasty Register.. BMC Musculoskelet Disord.

[CIT0009] Tarasevicius S, Kesteris U, Robertsson O, Smailys A, Janusonis V, Wingstrand H (2007). Introduction of total hip arthroplasty in Lithuania: results from the first 10 years.. Acta Orthop.

